# Mepolizumab in patients with severe asthma and blood eosinophil counts between 150 and 300 cells per µL: benefits at two years

**DOI:** 10.1183/23120541.01390-2024

**Published:** 2025-11-10

**Authors:** Giorgio W Canonica, Diego Bagnasco, Jason K Lee, Geoffrey Chupp, Florence Schleich, John J Oppenheimer, Lingjiao Zhang, Rafael Alfonso-Cristancho, Peter Howarth

**Affiliations:** 1 Personalized Medicine, Asthma and Allergy, Humanitas Clinical and Research Center, IRCCS, Rozzano, Italy; 2 Department of Biomedical Sciences, Humanitas University, Milan, Italy; 3 Allergy and Respiratory Diseases, IRCCS Policlinic San Martino, University of Genoa, Genoa, Italy; 4 Toronto Allergists, Toronto, Canada; 5 Evidence Based Medical Educator Inc., Toronto, Canada; 6 Department of Internal Medicine, Yale School of Medicine, New Haven, CT, USA; 7 GIGA Research Group, CHU of Liège, University of Liege, Liege, Belgium; 8 UMDNJ, Rutgers University School of Medicine, Newark, NJ, USA; 9 Biostatistics, GSK, Collegeville, PA, USA; 10 Global Real-World Evidence and Health Outcomes Research, GSK, Collegeville, PA, USA; 11 Global Medical Affairs, Specialty Medicine, GSK, London, UK

## Abstract

**Background:**

Although clinical trial evidence exists, there is limited awareness of the real-world effectiveness of mepolizumab in patients with severe asthma and blood eosinophil counts (BEC) ≥150–<300 cells·μL^−1^.

**Methods:**

REALITI-A, an international, prospective, single-arm, observational cohort study enrolled patients with severe asthma initiating mepolizumab. Outcomes assessed over 2 years pre- *versus* post-mepolizumab exposure included clinically significant exacerbations (CSEs), maintenance oral corticosteroid (mOCS) use, Asthma Control Questionnaire (ACQ)-5 scores and forced expiratory volume in 1 s (FEV_1_).

**Results:**

After 2 years of mepolizumab treatment, compared with pre-exposure, the proportion of patients with BEC ≥150–<300 cells·μL^−1^ (n=84) experiencing CSEs decreased from 95% to 63%, and the proportion experiencing exacerbations requiring hospitalisation or emergency department visits decreased from 43% to 19%. The rate of CSEs reduced from 4.53 to 1.47 per year (rate ratio 0.32, 95% CI 0.25, 0.41). After 2 years of mepolizumab exposure, the mean (95% CI) clinic pre-bronchodilator % predicted FEV_1_ was 80.1 (69.4, 90.7) compared with 62.6 (54.1, 71.1) at baseline (28% relative increase). The median average daily dose of mOCS decreased from 10.0 to 1.5 mg·day^−1^ (85% relative reduction from baseline); 44% of patients discontinued completely. The minimum clinically important difference in ACQ-5 (improvement ≥0.5) was achieved by 82% of patients, with a mean (95% CI) reduction of 1.76 (2.34, 1.19).

**Conclusions:**

These real-world findings provide evidence for the 2-year sustained benefit following initiation of mepolizumab in patients with severe asthma who have poor disease control and BEC ≥150–<300 cells·μL^−1^.

## Shareable abstract

**Patients with severe asthma and blood eosinophil counts ≥150–<300 cells·μL^−1^ showed reduced severe disease exacerbations and maintenance oral corticosteroid use with mepolizumab, whilst improving asthma control and lung function in routine clinical practice** https://bit.ly/40dTdHR

## Graphical abstract

**Figure:**
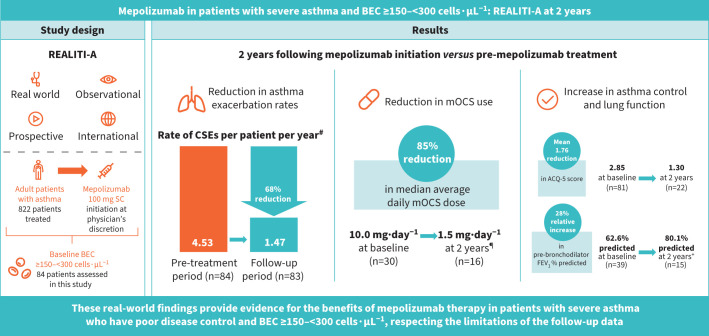
Overview of the study. This study was funded by GSK (204710); please refer to the text for additional disclosures. ^#^: analyses of annual exacerbation rates were performed using a generalised estimating equation model assuming a negative binomial distribution, with the covariate of treatment period pre- and post-exposure; ^¶^: assessed at Weeks 101–104; ^+^: assessed at Months 21–24. ACQ: Asthma Control Questionnaire; BEC: blood eosinophil count; CSE: clinically significant exacerbation; FEV_1_: forced expiratory volume in 1 s; mOCS: maintenance oral corticosteroid; SC: subcutaneous.

## Introduction

Severe asthma is characterised by frequent exacerbations despite the use of inhaled corticosteroids, long-acting β_2_-agonists or oral corticosteroids (OCS) [1–3]. Up to 80% of patients with severe asthma are likely to have an eosinophilic phenotype [4, 5]. The severity and burden of asthma can be predicted by the presence of, among other characteristics, an elevated blood eosinophil count (BEC); for example, in one large cohort study, at BEC cut-off values of >300 cells·µL^−1^, >400 cells·µL^−1^ and >500 cells·µL^−1^, the rate ratios (RR) for severe exacerbations were 1.29 (95% confidence interval (CI) 1.25, 1.33), 1.42 (1.36, 1.47) and 1.58 (1.50, 1.66), respectively [6].

Mepolizumab is a humanised anti-interleukin-5 monoclonal antibody approved for use as an add-on maintenance treatment in adults and children 6 years of age and older with severe asthma with an eosinophilic phenotype [7, 8]. The suitability of mepolizumab for treating patients with inadequately controlled severe asthma despite standard of care treatment, who have an eosinophilic phenotype, is well established [9–22].

The appropriate threshold at which to recommend mepolizumab treatment remains unclear for the BEC criterion, with some sources suggesting an optimal starting point of ≥150 cells·μL^−1^ [23, 24]. Data from Phase III clinical studies demonstrated the benefits of mepolizumab in patients with severe asthma with BEC ≥150 cells·μL^−1^ pre-exposure [9, 11, 17, 18]. However, this categorisation included patients with BEC above this threshold, with the majority of patients having very high BEC (∼300  cells·μL^−1^ or more) pre-exposure [11, 17, 18, 23]. Even though *post hoc* analyses of these clinical studies in patients with a pre-exposure BEC ≥150–<300 cells·μL^−1^ gave positive results [23, 25], support from real-world studies is needed.

In contrast to randomised controlled trials (RCTs) [9, 11, 17, 18], real-world studies offer results on effectiveness from a diversified patient population, owing to their less restrictive inclusion criteria. These complementary data are essential to validate the findings of RCTs. REALITI-A was a 2-year, international, prospective study assessing the real-world clinical effectiveness of mepolizumab in patients with severe asthma [13, 15, 19, 26]. Preliminary results from REALITI-A at 1 year showed that mepolizumab treatment reduces the rate of clinically significant exacerbations (CSEs) across all pre-exposure BEC levels (including the ≥150–<300 cells·μL^−1^ range) [13].

This *post hoc* analysis of 2-year data from the REALITI-A study aimed to comprehensively assess the real-world effectiveness of mepolizumab in patients with severe asthma and baseline BEC ≥150–<300 cells·μL^−1^.

## Methods

### Study design and treatment

The REALITI-A study (GSK ID: 204710) was an international, prospective, single-arm, observational cohort study enrolling patients diagnosed with severe eosinophilic asthma and newly prescribed mepolizumab treatment (100 mg subcutaneously) based on a physician's decision. The detailed study design and analyses at 1 and 2 years have been previously published [13, 15, 19, 26]. Patients were enrolled between December 2016 and October 2019. The index date was the date of first mepolizumab administration. The pre-treatment period was defined as 365 days prior to enrolment plus the length of the run-in period (if applicable). The post-mepolizumab period comprised the 365 days following the index date.

### Patients

Inclusion and exclusion criteria for REALITI-A are described in detail elsewhere [13, 15, 19, 26]. Eligible patients were aged 18 years or older with a current diagnosis of asthma, a physician's decision to initiate mepolizumab treatment and relevant medical records for at least 12 months before enrolment. Prior use of other biological medications was permitted, but those who received mepolizumab in the year before enrolment or participated in an interventional clinical trial within the year before enrolment were excluded. Patients were recruited from 84 centres in seven countries.

### End-points and assessments

All outcomes were assessed in patients with baseline BEC ≥150–<300 cells·μL^−1^. The primary outcome was the rate of CSEs between the pre-treatment and post-mepolizumab periods. CSEs were defined as a deterioration in asthma requiring the use of systemic corticosteroids (SCS) and/or an emergency department (ED) visit and/or hospitalisation. The use of SCS was defined as OCS for ≥3 days or a single intravenous or intramuscular administration of SCS due to worsening asthma symptoms; for patients on maintenance OCS (mOCS), use of SCS was defined as a two-fold or greater increase in the existing maintenance dose for ≥3 days [13, 15, 19, 26].

Secondary objectives compared the following outcomes in the same periods: rate of asthma exacerbations requiring hospitalisation and/or ED visit or hospitalisation only, the proportion of patients without CSEs and the proportion of patients achieving >0–<50% and 50–100% reductions in CSE rates.

Other outcomes included the pre-bronchodilator forced expiratory volume in 1 s (FEV_1_) during the post-mepolizumab period. The proportion of patients who achieved an increase in FEV_1_ ≥100 mL from baseline was assessed for patients with FEV_1_ data available at baseline that had reported a new FEV_1_ during the last 3-month reporting period. Changes from baseline in mOCS daily dose and total OCS daily dose (in those requiring mOCS) were also assessed. The total OCS daily dose included both mOCS and rescue doses; both mOCS and total OCS doses were expressed as prednisone-equivalent doses (mg·day^−1^). The baseline for the mOCS and total OCS dose analyses was defined as the 28 days preceding the index date. The change from baseline in Asthma Control Questionnaire-5 (ACQ-5) score during the post-mepolizumab period was also assessed. ACQ-5 scores [27] were collected on a voluntary basis at baseline and no more frequently than every 3 months at usual asthma healthcare visits during the post-mepolizumab period. The proportion of patients achieving the minimal clinically important difference (MCID) in ACQ-5 scores (≥0.5 point improvement [28]) was assessed for the patients with ACQ-5 data available at baseline that had reported a new ACQ-5 score during the last 3-month reporting period. For ACQ-5 and FEV_1_, baseline was defined as the value collected at the index date or the nearest historical value within 90 days prior to the index date.

These outcomes were also reported for the subset of patients with baseline BEC ≥300–<500 cells·μL^−1^ in the REALITI-A study, as a comparison with the primary patient population (baseline BEC ≥150–<300 cells·μL^−1^).

### Sample size and statistical analysis

Descriptive statistics, including mean (standard deviation (sd)/95% CI), median (first quartile (Q1) to third quartile (Q3)/95% CI) and n (%) were used for continuous variables and for ACQ-5 and FEV_1_ responder analyses. Relative change from baseline/pre-treatment at a particular timepoint was calculated as the difference between the value at the timepoint and the value at baseline/pre-treatment, divided by the value at baseline/pre-treatment; a negative (*versus* positive) value was labelled as a “reduction” (*versus* “increase”). Analyses of annual exacerbation rates were performed using a generalised estimating equation (GEE) model assuming a negative binomial distribution, with the covariate of treatment period pre- and post-exposure. The variance of the mean estimate was corrected for within-patient correlation. The logarithm of time was used as an offset variable. Owing to model-estimated rates, the pre-exposure rate may have differed between post-exposure sections and estimands. Within the subset of patients providing baseline BEC results, annual rates of CSEs were compared between the pre-treatment and the post-mepolizumab periods for patients stratified by thresholds of baseline BECs (as continuous variables) using the aforementioned model with covariates of period (pre-treatment, post-mepolizumab), baseline BEC and baseline BEC by period interaction. For the proportion of patients with zero exacerbations, zero exacerbations requiring hospitalisation, and zero exacerbations requiring hospitalisation or ED visits, a logistic regression model was performed separately for each subgroup level, for comparisons between pre- and post-exposure *via* GEE with covariates of time period (pre-, post-exposure) by inclusion of the REPEATED statement of the GENMOD procedure. Analysis for the subgroup was performed if 10 or more patients with analysable data were available. Mean change from baseline in ACQ-5 score was analysed using a mixed model repeated measures approach, with covariates of timepoint, country, exacerbations during the pretreatment period and use of mOCS at enrolment.

This study was conducted in accordance with the Declaration of Helsinki. The study protocol, amendments, informed consent forms and other information requiring pre-approval were reviewed and approved by a national, regional, or investigational centre ethics committee or institutional review board.

## Results

### Patients

Of the 822 REALITI-A patients, 84 had a baseline BEC ≥150–<300 cells·μL^−1^. Of these 84 patients, 61% were female adults under 64 years of age from Italy and the UK; 74% were between 18 and 64 years of age, while 26% were between 65 and 84 years of age; 88% were white or Caucasian (table 1). Of these 84 patients, 56% completed the study without discontinuation of mepolizumab (table S1). The mean BEC at baseline was 211 cells·μL^−1^, with a mean highest count of 378 cells·µL^−1^ in the 12 months pre-enrolment. After 2 years of mepolizumab exposure, the mean BEC was 87 cells·μL^−1^, which corresponded to a mean ratio to baseline of 0.38.

**TABLE 1:** Patient demographics and clinical characteristics during the pre-mepolizumab period (baseline)

Characteristics	Patients with baseline^#^ BEC ≥150–<300 cells·μL^−1^
**Patients, n**	84
**Sex, n (%)**	
Female	51 (61)
Male	33 (39)
**Age years** ^¶^	
Mean±sd	54.3±14.2
Min–max	19–83
**Age group years,**^¶^ **n (%)**	
18–64 65–84	62 (74) 22 (26)
**Ethnicity, n (%)**	
Hispanic or Latino	7 (8)
Not Hispanic or Latino	77 (92)
**Race, n (%)**	
Asian, Native Hawaiian or Other Pacific Islander	6 (7)
Black or African American	4 (5)
White or Caucasian	74 (88)
**Country, n (%)**	
Canada	4 (5)
EU (excluding Italy)	11 (13)
Italy	31 (37)
UK	26 (31)
USA	12 (14)
**BMI kg·m^−2^**	
Mean±sd	29.60±7.0
Min–max	18.2–54.5
**Asthma duration years**	n=82
Mean±sd	22.8±17.1
Min–max	0.0–63.2
**Asthma duration years, n (%)**	n=82
0 to <10 years	25 (30)
≥10 to <20 years	17 (21)
≥20 years	40 (49)
**Atopic status, n (%)**	
Yes	47 (56)
No	4 (5)
Unknown	33 (39)
**Total IgE KU·L^−1^**	n=72
Geometric mean±sd log	124.5±1.6
***F*_ENO_ ppb**	n=34
Mean±sd	41.1±39.2
Min–max	5.0–154.0
**Ever treated with omalizumab, n (%)**	
Yes	15 (18)
No	69 (82)
**Duration of omalizumab treatment months**	n=15
Mean±sd	37.4±33.9
Min–max	1–116
**Primary reason for treating with mepolizumab, n (%)**	
Reduce exacerbations	38 (51)
Reduce burden of oral corticosteroids	12 (16)
Improve asthma symptoms	19 (25)
Improve quality of life	6 (8)
Improve lung function	0 (0)
**Clinically significant exacerbations** ^+^	
Mean±sd	4.6±4.5
**Clinically significant exacerbations,**^+^ **n (%)**	
0	4 (5)
1	12 (14)
≥2	68 (81)
**Exacerbations requiring ED visits and/or hospitalisation** ^+^	
Mean±sd	1.3±2.9
**Exacerbations requiring ED visits and/or hospitalisation,**^+^ **n (%)**	
0	48 (57)
1	14 (17)
≥2	22 (26)
**Exacerbations requiring hospitalisation** ^+^	
Mean±sd	0.6±1.3
**Exacerbations requiring hospitalisation,**^+^ **n (%)**	
0	58 (69)
1	13 (15)
≥2	13 (15)

BEC: blood eosinophil count; BMI: body mass index; IgE: immunoglobulin E; *F*_ENO_: fractional exhaled nitric oxide; ppb: parts per billion; ED: emergency department. ^#^: baseline was defined as the value that was collected on the index date, or the nearest historical value within the 90 days prior to the index date, and values during the post-mepolizumab period were collected no more frequently than monthly at the usual asthma healthcare visits; ^¶^: age was imputed when full date of birth was not provided; ^+^: in the 12 months pre-enrolment.

The mean±sd duration of asthma was 22.8±17.1 years, with 44% of patients having disease duration exceeding 25 years. At the start of the REALITI-A study, omalizumab was the only available biologic treatment for severe asthma; only 18% of patients had ever been treated with omalizumab, with a mean±sd treatment duration of 37.4±33.9 months. The primary reason (51% of patients) for initiating mepolizumab was to reduce exacerbations, according to the healthcare practitioner. Other reasons included improving asthma symptoms (25%), reducing the burden of oral corticosteroids (16%) and improving quality of life (8%).

### Exacerbations

**FIGURE 1:**
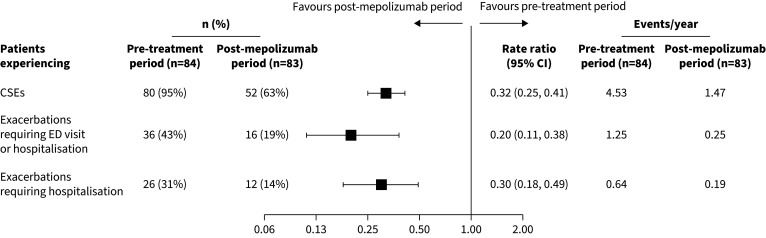
Proportion of patients experiencing clinically significant exacerbations (CSEs), exacerbations requiring emergency department (ED) visits or hospitalisation, and exacerbations requiring hospitalisation and comparison of yearly rates, pre- *versus* post-mepolizumab exposure. Analyses of annual exacerbation rates were performed using a generalised estimating equation (GEE) model assuming a negative binomial distribution, with the covariate of treatment period pre- and post-exposure.

**FIGURE 2:**
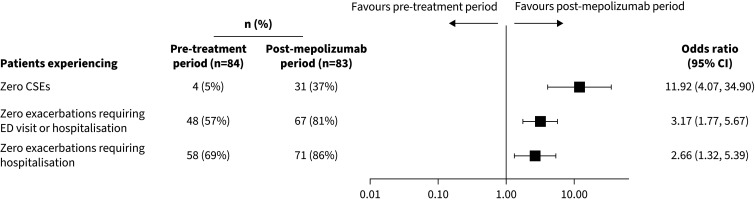
Proportion of patients experiencing zero clinically significant exacerbations (CSEs), zero exacerbations requiring emergency department (ED) visit or hospitalisation, and zero exacerbations requiring hospitalisation, pre- *versus* post-mepolizumab exposure. Analyses of annual exacerbation rates were performed using a generalised estimating equation (GEE) model assuming a negative binomial distribution, with the covariate of treatment period pre- and post-exposure.

In the 12 months pre-enrolment, 37% of patients experienced ≥5 CSEs, with an overall mean±sd of 4.6±4.5. Patients experienced a mean±sd of 1.3±2.9 exacerbations requiring hospitalisations or ED visits and 0.6±1.3 exacerbations requiring hospitalisations. The proportion of patients experiencing CSEs reduced from 95% pre-mepolizumab exposure (80 out of 84) to 63% post-mepolizumab exposure (52 out of 83). Additionally, the proportion of patients experiencing exacerbations requiring hospitalisation or ED visits decreased from 43% (36 out of 84) to 19% (16 out of 83). The CSE rate decreased from 4.53 per year pre-exposure to 1.47 per year post-exposure (RR 0.32, 95% CI 0.25–0.41) (figure 1). The rate of exacerbations requiring hospitalisation or ED visits also decreased, from 1.25 to 0.25 per year (RR 0.20, 95% CI 0.11, 0.38). The rate of exacerbations requiring hospitalisation decreased from 0.64 to 0.19 per year (RR 0.30, 95% CI 0.18, 0.49). The proportion of patients experiencing zero CSEs grew more than six-fold compared with the pre-exposure period, increasing from 5% (4 out of 84) to 37% (31 out of 83) (odds ratio (OR) 11.92, 95% CI 4.07, 34.90) (figure 2). The proportion of patients experiencing zero exacerbations requiring hospitalisation or ED visits increased from 57% (48 out of 84) to 81% (67 out of 83) (OR 3.17, 95% CI 1.77, 5.67). The proportion of patients experiencing zero exacerbations requiring hospitalisation increased from 69% (58 out of 84) to 86% (71 out of 83) (OR 2.66, 95% CI 1.32, 5.39). After 2 years of mepolizumab exposure, 69% of patients achieved a ≥50% reduction in CSEs, 37% achieved a ≥50% reduction in exacerbations requiring hospitalisations or ED visits, and 28% achieved a ≥50% reduction in exacerbations requiring hospitalisations, compared with pre-exposure (table 2).

**TABLE 2:** Proportion of patients achieving reduction in rate of clinically significant exacerbations (CSEs) after 2 years post-mepolizumab exposure, compared with the pre-exposure period

	Patients with baseline BEC ≥150–<300 cells·μL^−1^
**Patients, n**	83^#^
**CSEs, 0–24 months, n (%)**	
50–100% reduction	57 (69)
>0–<50% reduction	16 (19)
No change/increase	10 (12)
**Exacerbations requiring hospitalisations or ED visits, 0–24 months, n (%)**	
50–100% reduction	31 (37)
>0–<50% reduction	4 (5)
No change/increase	48 (58)
**Exacerbations requiring hospitalisations, 0–24 months, n (%)**	
50–100% reduction	23 (28)
>0–<50% reduction	3 (4)
No change/increase	57 (69)

BEC: blood eosinophil count; ED: emergency department. ^#^: patients with analysable data for both historical exacerbations and post-exposure exacerbations.

### Lung function

**FIGURE 3:**
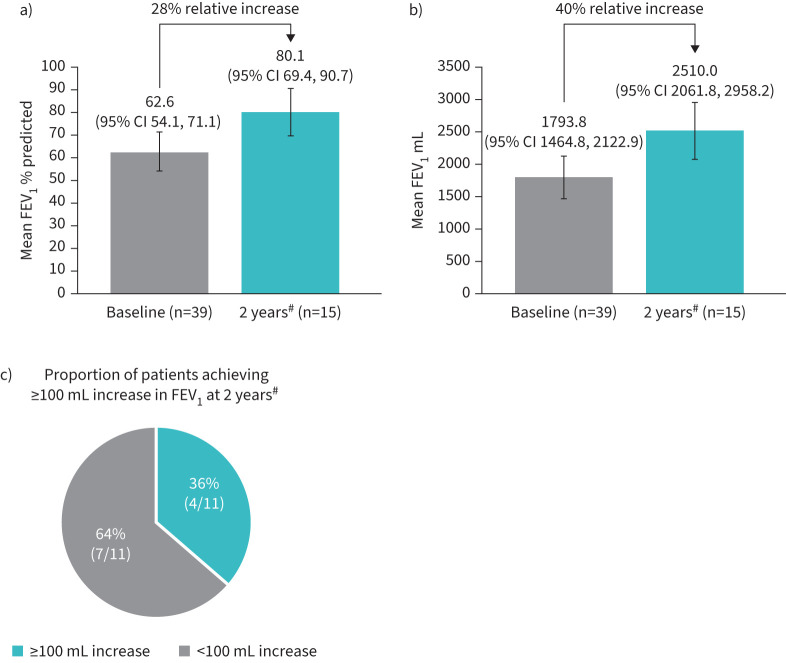
Clinic pre-bronchodilator forced expiratory volume in 1 s (FEV_1_) assessed by a) mean % predicted FEV_1_ at baseline and 2 years post-mepolizumab exposure, b) mean absolute FEV_1_ at baseline and 2 years post-mepolizumab exposure, and c) proportion of patients achieving ≥100 mL increase in absolute FEV_1_ at 2 years post-mepolizumab exposure. ^#^: assessed at Months 21–24.

Mean (95% CI) clinic pre-bronchodilator % predicted FEV_1_ increased by 28% relative to baseline, rising from 62.6 (54.1, 71.1) at baseline to 80.1 (69.4, 90.7) at the end of the study (figure 3a). Mean (95% CI) absolute clinic pre-bronchodilator FEV_1_ increased by 40% relative to baseline, rising from 1793.8 (1464.8, 2122.9) mL at baseline to 2510.0 (2061.8, 2958.2) mL at the end of the study (figure 3b). Of 11 patients with available pre-bronchodilator FEV_1_ data at both baseline and 2 years, four patients (36%) achieved an improvement of ≥100 mL in FEV_1_ (figure 3c).

### OCS use

**FIGURE 4:**
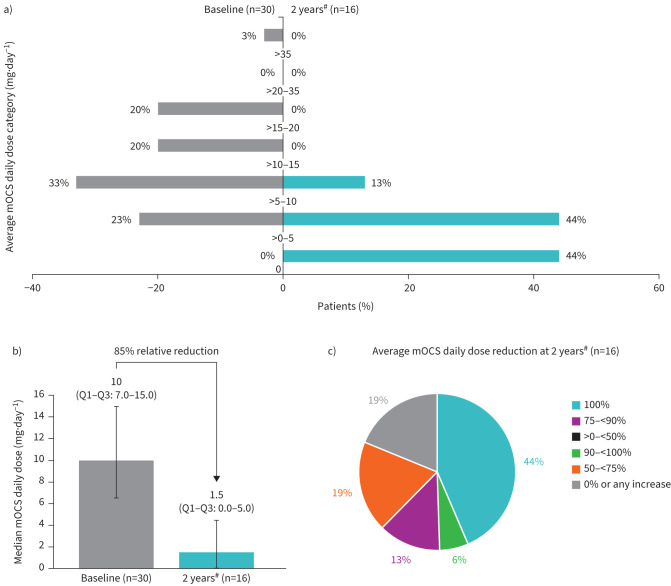
Average maintenance oral corticosteroid (mOCS) daily dose assessed by a) dose category at baseline and 2 years post-mepolizumab exposure, b) median (Q1–Q3) dose at baseline and 2 years post-mepolizumab exposure, and c) % reduction dose category at 2 years post-mepolizumab exposure. Q1: first quartile; Q3: third quartile. ^#^: assessed at Weeks 101–104.

At baseline, 43% of the 30 patients receiving mOCS had an average daily mOCS dose >10 mg·day^−1^. After 2 years of mepolizumab exposure, of 16 patients with available data, 44% (7 out of 16) were no longer receiving mOCS and 56% (9 out of 16) were receiving doses of 10 mg·day^−1^ or less (figure 4a). The median average daily dose of mOCS decreased from 10.0 mg·day^−1^ at baseline to 1.5 mg·day^−1^ after 2 years of mepolizumab exposure, a relative reduction of 85% (figure 4b), which was a continued improvement compared with the 60% relative reduction observed after 1 year (supplementary figure S1). Additionally, the median average daily dose of total OCS decreased from 14.5 mg·day^−1^ at baseline to 1.8 mg·day^−1^ after 2 years, a relative reduction of 88%. After 2 years of mepolizumab exposure, 44% (seven out of 16) of patients were no longer receiving mOCS (100% reduction from baseline in average daily mOCS dose), 6% (one out of 16) achieved a 90–<100% reduction from baseline in average daily mOCS dose, 13% (two out of 16) achieved a 75–<90% reduction, 19% (three out of 16) achieved a 50–<75% reduction, and 19% (three out of 16) experienced either no change or an increase in their mOCS dose (figure 4c). After 2 years of mepolizumab exposure, the median (95% CI) % reduction from baseline in average daily mOCS dose was 86.7% (51.5–100), which was an improvement compared with the % reduction of 51.5% (0.0–100) at 1 year (supplementary figure S2).

### Asthma control

**FIGURE 5:**
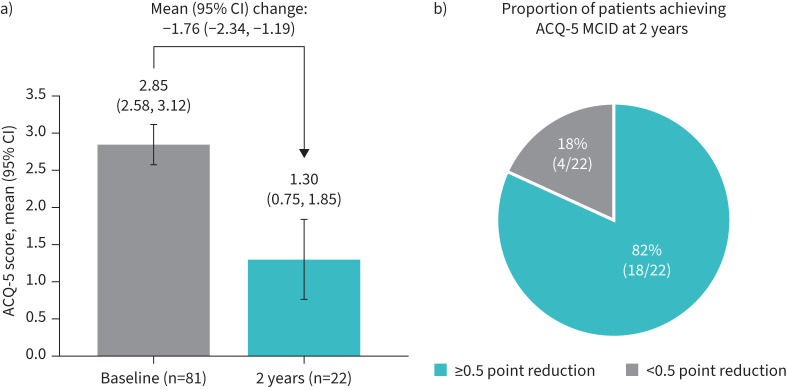
Asthma control assessed by a) mean Asthma Control Questionnaire-5 (ACQ-5) score at baseline and 2 years post-mepolizumab exposure, and b) proportion of patients achieving ACQ-5 minimal clinically important difference (MCID) (≥0.5 point improvement) at 2 years post-mepolizumab exposure.

Mean (95% Cl) ACQ-5 score was 2.85 (2.58, 3.12) at baseline, and was reduced to 1.30 (0.75, 1.85) after 2 years of mepolizumab exposure (mean (95% CI) change from baseline: −1.76 (−2.34,  −1.19)) (figure 5a). More than three-quarters (82%, 18 out of 22) of patients with available ACQ-5 data at both baseline and 2 years achieved the MCID in ACQ-5 improvement at 2 years (figure 5b).

### Baseline eosinophil categories

After 2 years of mepolizumab exposure, compared with patients with baseline BEC ≥150–<300 cells·μL^−1^ (n=83), patients with baseline BEC ≥300–<500 cells·μL^−1^ (n=154) experienced similar reductions in CSE rates, greater reductions in daily mOCS dose, similar improvements in ACQ-5 scores and greater improvements in clinic pre-bronchodilator FEV_1_ (supplementary table S2).

## Discussion

The efficacy of mepolizumab in patients with severe asthma and BEC ≥150–<300 cells·μL^−1^ has been well established in clinical trial settings, but real-world evidence with long-term outcomes is needed. This *post hoc* analysis of the REALITI-A patient population assessed the real-world effectiveness of mepolizumab in patients with severe asthma and BEC ≥150–<300 cells·μL^−1^ at 2 years. The results demonstrated reductions in the frequency of CSEs and exacerbations requiring hospitalisation and/or ED visits. There was a decrease in mOCS use, along with an improvement in asthma control and lung function over 2 years of treatment in this patient subgroup. These findings are in alignment with the overall REALITI-A patient population as previously published [26].

Several RCTs have proven the efficacy of mepolizumab in reducing exacerbations among patients with BEC >150 cells·μL^−1^ at baseline [23, 25]. An in-depth analysis of these studies found the pre-treatment morbidity among patients with BEC ≥150–<300 cells·μL^−1^ was comparable to that among patients with BEC ≥300 cells·μL^−1^, with similar levels of healthcare resource utilisation and exacerbations in the year prior to each study [25]. Furthermore, the DREAM, MENSA and MUSCA trials, respectively, reported 49%, 36% and 27% reduction in CSEs post-mepolizumab among patients with baseline BEC ≥150–<300 cells·μL^−1^ [25]. Additionally, the SIRIUS study found that more patients in the mepolizumab group reduced their SCS dose compared with placebo, with an OR (95% CI) of 2.03 (0.53, 7.75) in patients with BEC ≥150–<300 cells·μL^−1^ at baseline, and 1.79 (0.71, 4.52) in those with BEC ≥300 cells·μL^−1^ [25]. Together, these results suggested a favourable risk–benefit profile for mepolizumab in patients with baseline BEC ≥150–<300 cells·μL^−1^. Therefore, solely limiting the eligibility for mepolizumab treatment to patients with BEC ≥300 cells·μL^−1^ may lead to appropriate patients missing out on the benefits of mepolizumab treatment. The findings of our *post hoc* analysis of real-world REALITI-A data were consistent with these RCT results, confirming that a threshold of BEC ≥150 cells·μL^−1^ would benefit more patients in real-world practice.

A review of country-specific prescription criteria for biologics, including mepolizumab, was carried out in April 2021 by Porsbjerg and colleagues [29]. Out of the 28 countries evaluated, nearly two-thirds used a threshold of BEC >300 cells·μL^−1^ for eligibility for starting mepolizumab treatment in the past 12 months (or ever in the past) preceding the study, and in Spain the threshold value was BEC >500 cells·μL^−1^ for mepolizumab. Additional restrictions may apply for treatment eligibility as well, with approximately half of the countries requiring two or more exacerbations in the year preceding a biologic prescription [29]. These findings suggest that, despite the growing body of evidence supporting the use of mepolizumab in patients with BEC ≥150–<300 cells·μL^−1^ in both clinical and real-world settings, current eligibility criteria prevent many patients who might clearly benefit from mepolizumab from receiving the drug. These patients continue to require frequent courses of OCS, which carry long-term, potentially irreversible, harm.

The current study demonstrated that outcome measures such as CSEs, lung function, mOCS use and asthma control all improved with mepolizumab. Improvements in these measures are usually the four critical criteria used for defining clinical remission in asthma, although with varying timescales and end-points between studies [30, 31]. Clinical remission is fast becoming an ambitious and achievable target for some patients, even those with severe asthma, in part due to the recent development of precision therapies [32, 33]. The Global Initiative for Asthma 2024 report highlights that the use of standardised criteria and tools for the measurement of clinical remission may assist in developing new approaches to asthma prevention and management [2, 25, 30, 32, 33]. The current study highlights that patients with BEC ≥150–<300 cells·μL^−1^ may need further investigation as a suitable population for clinical remission attainment.

The strengths and limitations of the REALITI-A study have been described [13, 15, 19, 26]. Briefly, the strengths of this study include its real-world setting, which enhances the generalisability of the findings to clinical practice. Unlike RCTs with strict inclusion criteria, REALITI-A reflects a broader patient population than typically encountered in clinical practice. Limitations include missing information, inadequate recording of events, possibility of self-medication and limited control over patient behaviour, which are common limitations of most real-world studies. In this subgroup analysis, low data availability at 2 years led to small sample sizes, especially for results related to mOCS use, asthma control and lung function. Nonetheless, this study's findings corroborated those observed in the whole REALITI-A population at 2 years and are relevant to drive further research of the benefits of mepolizumab in patients with BEC ≥150–<300 cells·μL^−1^.

In conclusion, this analysis supports the real-world effectiveness of mepolizumab in severe asthma for patients with BEC ≥150–<300 cells·μL^−1^, as demonstrated through improvements in exacerbations, mOCS sparing, asthma control and lung function at 2 years.

## Data Availability

Please refer to GSK weblink to access GSK's data sharing policies and as applicable seek anonymised subject level data *via* the link https://www.gsk-studyregister.com/en/.

## Supplementary material

**Please note:** supplementary material is not edited by the Editorial Office, and is uploaded as it has been supplied by the author. Supplementary material 01390-2024.SUPPLEMENT Supplementary material 01390-2024.SUPPLEMENT Supplementary material 01390-2024.SUPPLEMENT2 Supplementary material 01390-2024.SUPPLEMENT2 Supplementary material 01390-2024.SUPPLEMENT3 Supplementary material 01390-2024.SUPPLEMENT4
