# Supplementary figures and images for: Mepolizumab in patients with severe asthma and blood eosinophil counts between 150 and 300 cells per µL: benefits at two years
Source: ERJ Open Res. 2025 Nov 10;11(6):01390-2024. doi: 10.1183/23120541.01390-2024 (PMC12598589; doi:10.1183/23120541.01390-2024)

a)

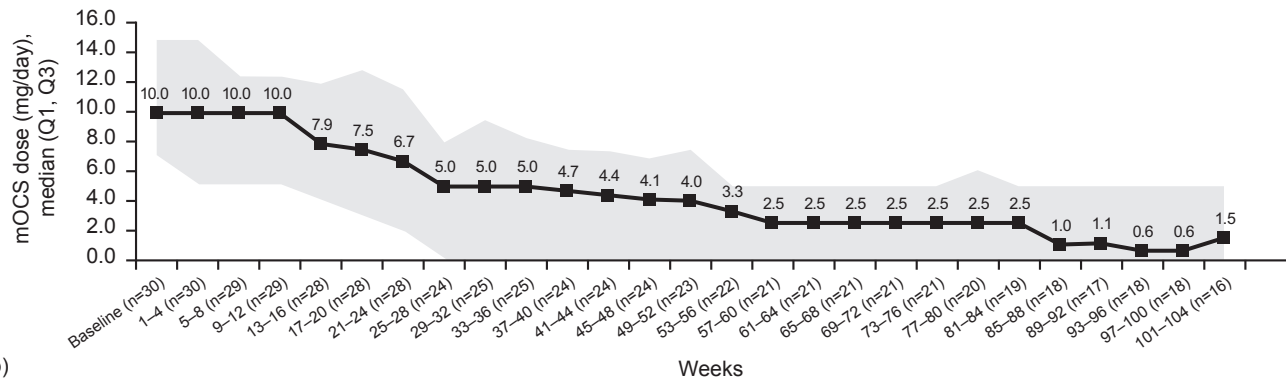

b)

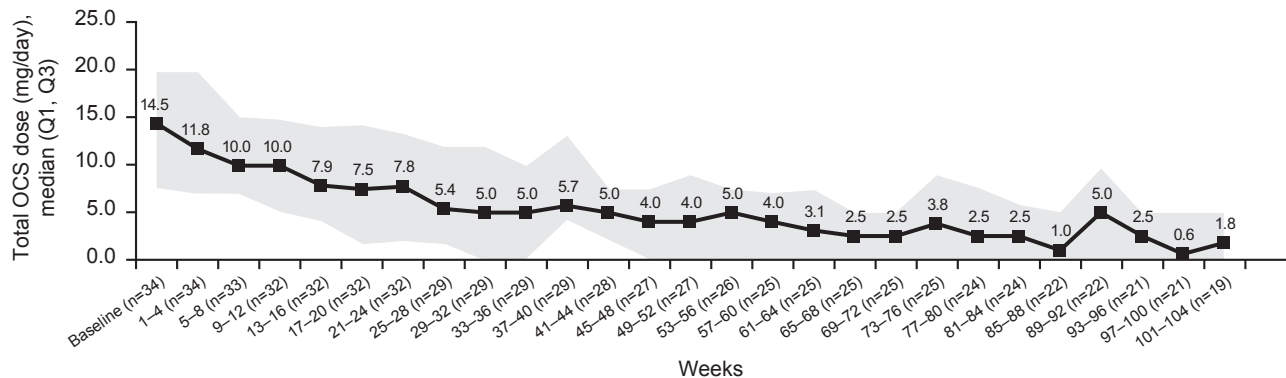

Supplement: Supplementary file 1 [file 01390-2024.SUPPLEMENT.pdf]

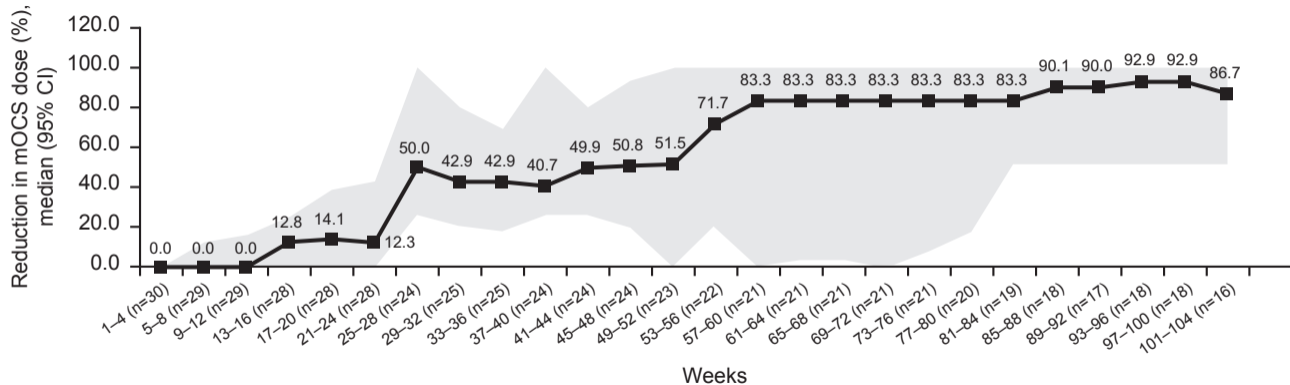

Supplement: Supplementary file 3 [file 01390-2024.SUPPLEMENT2.pdf]
