# Supplementary material for: Mepolizumab in patients with severe asthma and blood eosinophil counts between 150 and 300 cells per µL: benefits at two years
Source: ERJ Open Res. 2025 Nov 10;11(6):01390-2024. doi: 10.1183/23120541.01390-2024 (PMC12598589; doi:10.1183/23120541.01390-2024)
Supplement: Supplementary file 2 [file 01390-2024.SUPPLEMENT.pptx]

## Slide 1
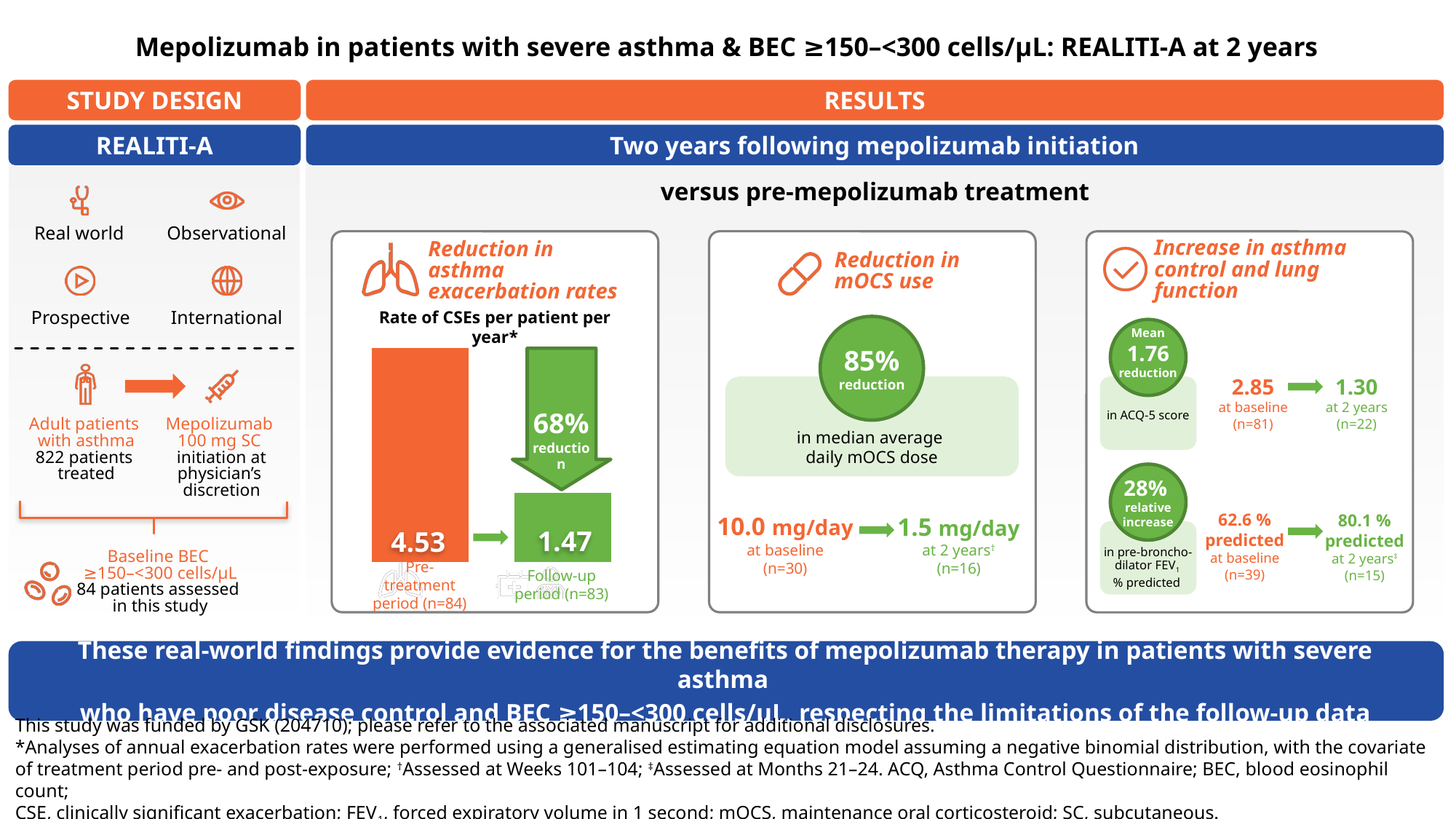

Mepolizumab in patients with severe asthma & BEC ≥150–<300 cells/µL: REALITI-A at 2 years
STUDY DESIGN
RESULTS
REALITI-A
Two years following mepolizumab initiation
versus pre-mepolizumab treatment
Real world
Observational
Reduction in asthma exacerbation rates
### Chart
| Category | Series 1 | Column1 | Column2 |
|---|---|---|---|
| pre | 4.53 | None | None |
| post | 1.47 | None | None |Rate of CSEs per patient per year*
1.47
4.53
Pre-treatment period (n=84)
Follow-up period (n=83)
68%
reduction
Reduction in
mOCS use
 85%
reduction
in median average
daily mOCS dose
10.0 mg/day
at baseline
(n=30)
1.5 mg/day
at 2 years†
(n=16)
Increase in asthma control and lung function
Mean
1.76
reduction
2.85
at baseline
(n=81)
1.30
at 2 years
(n=22)
in ACQ-5 score
28%
relative
increase
62.6 % predicted
at baseline
(n=39)
80.1 % predicted
at 2 years‡
(n=15)
in pre-broncho-dilator FEV1
% predicted
Prospective
International
Adult patients
with asthma
822 patients
treated
Mepolizumab
100 mg SC
initiation at physician’s
discretion
Baseline BEC
≥150–<300 cells/μL
84 patients assessed
in this study
These real-world findings provide evidence for the benefits of mepolizumab therapy in patients with severe asthma
who have poor disease control and BEC ≥150–<300 cells/μL, respecting the limitations of the follow-up data
This study was funded by GSK (204710); please refer to the associated manuscript for additional disclosures.
*Analyses of annual exacerbation rates were performed using a generalised estimating equation model assuming a negative binomial distribution, with the covariate of treatment period pre- and post-exposure; †Assessed at Weeks 101–104; ‡Assessed at Months 21–24. ACQ, Asthma Control Questionnaire; BEC, blood eosinophil count;
CSE, clinically significant exacerbation; FEV1, forced expiratory volume in 1 second; mOCS, maintenance oral corticosteroid; SC, subcutaneous.
